# Perception on aggregation induced multicolor emission and emission centers in carbon nanodots using successive dilution, anion exchange chromatography, and multi-way statistics

**DOI:** 10.1038/s41598-021-93212-w

**Published:** 2021-07-07

**Authors:** Mohsen Kompany-Zareh, Saeed Bagheri

**Affiliations:** 1 Department of Chemistry, Institute for Advanced Studies in Basic Sciences, 45137-66731 Zanjan, Iran; 2 Trace Analysis Research Centre, Department of Chemistry, Dalhousie University, P.O. Box 15000, Halifax, NS B3H 4R2 Canada

**Keywords:** Analytical chemistry, Infrared spectroscopy, Cheminformatics, Optical materials

## Abstract

Exploration in the way of understanding the optical behavior and structure of carbon nanodots has been increased due to their vast application. Their emission dependency on excitation wavelengths is the more prevalent and controversial subject. In this report we considered the optical structure of hydrothermally synthesized carbon nanodots using citric acid and 2,3-diaminopyridine as precursors. The presence of different emission centers experimented through anion exchange chromatography which resulted in fractions with more unique optical structures. The quantum confinement effect and energy exchange between different types of carbon nanodots, due to aggregation in higher concentration levels, was studied applying a stepwise dilution experiment. Analysis of the experimental data was done through the parallel factor analysis and the trajectory pattern recognition which resolved more about optical interactions and the presence of different emission centers in different particles. Results from infrared spectroscopy confirmed the dominating density of carboxyl functional groups on the nanodots with negative surface charges and higher influence of amine groups on dots with positive surface charges.

## Introduction

Since its emersion, fluorescence carbon nanodots (CND) entice the subject of material science, due to their exceptional optical and electronic properties, the availability of precursor materials, and ease of their synthesis^1–10^. In some cases, CNDs are considered among the quantum dots (QD) which are mainly synthesized by the heavy elements^3,6,9^. The quantum confinement effect is the signature feature of QDs which relates the emission band to their size, but the CNDs do not exhibit this feature in the same way and reasons^1,3,4,11–13^. The lack of this feature in CNDs illustrates that their optical characteristics are coming from some emission centers rather than the whole particle. In another definition, CNDs are spherical or nearly spherical carbon nanoparticles whose surfaces are stabilized by some functional and optically active groups^1,3,7,10–12,14–22^. On the other hand, the quantum confinement effect in CNDs could be viewed through the aggregation of different particles. The aggregation of different particles leads to the closeness of optical states which leads to a redshift of emission wavelengths^12–14,21,23–30^. Photoluminescence (PL) in CNDs is mainly dependent on the excitation wavelengths which is in contrast to the QDs. This dependency is mainly due to the existence of different luminescence centers which each have a different excitation pattern^1,3,6,8,10,15,20,22,31–36^. The extent of precursor materials for the synthesis of CNDs leads to different types of CNDs and optical characteristics^1–3,10,11^. The vast applicability of CNDs and their complicated optical behavior calls the researchers to the in-depth characterization of the optical nature. Different synthesis conditions^4,5,11,16,20,37^, solvent effect^11,12,19,32^, dilution effect^13,14,21,25,26,28–30^, solid-state^14,24^ and considering molecular by-products^1,38–42^ are some instances of the various subjects of CNDs considering by scientists. Here through changing the concentration in an evolutionary manner and separation of different species of CNDs along with the in-detail data analysis, we are trying to gain more information on optical characteristics of CNDs.

## Results

Carbon dot mixtures are amazing combinations of interacting particles with a variety of optical and surface characteristics. Using two simple experiments of ionic chromatographic separation and dilution, the goal in this report is the spectral investigation of separated carbon dots among fractions and interacting carbon dots in diluted mixtures.

### Anion exchange chromatography (AEC)

Particles with a higher number of negative charges show stronger interaction with the stationary phase, slower migration in the column, and higher retention times. For each elution sample, an excitation-emission matrix (EEM) spectral map was measured to track the PL changes of CNDs through separation by the column (Figure S2). The newly synthesized mixture of CNDs was fractionated into forty elution samples, from which 33 involved meaningful PL characteristics. The increase in the ionic strength of the mobile phase was initiated from the fifteenth elution sample and the first fourteen elution samples were eluted without substantial retention by the stationary phase.

**Figure 1:**
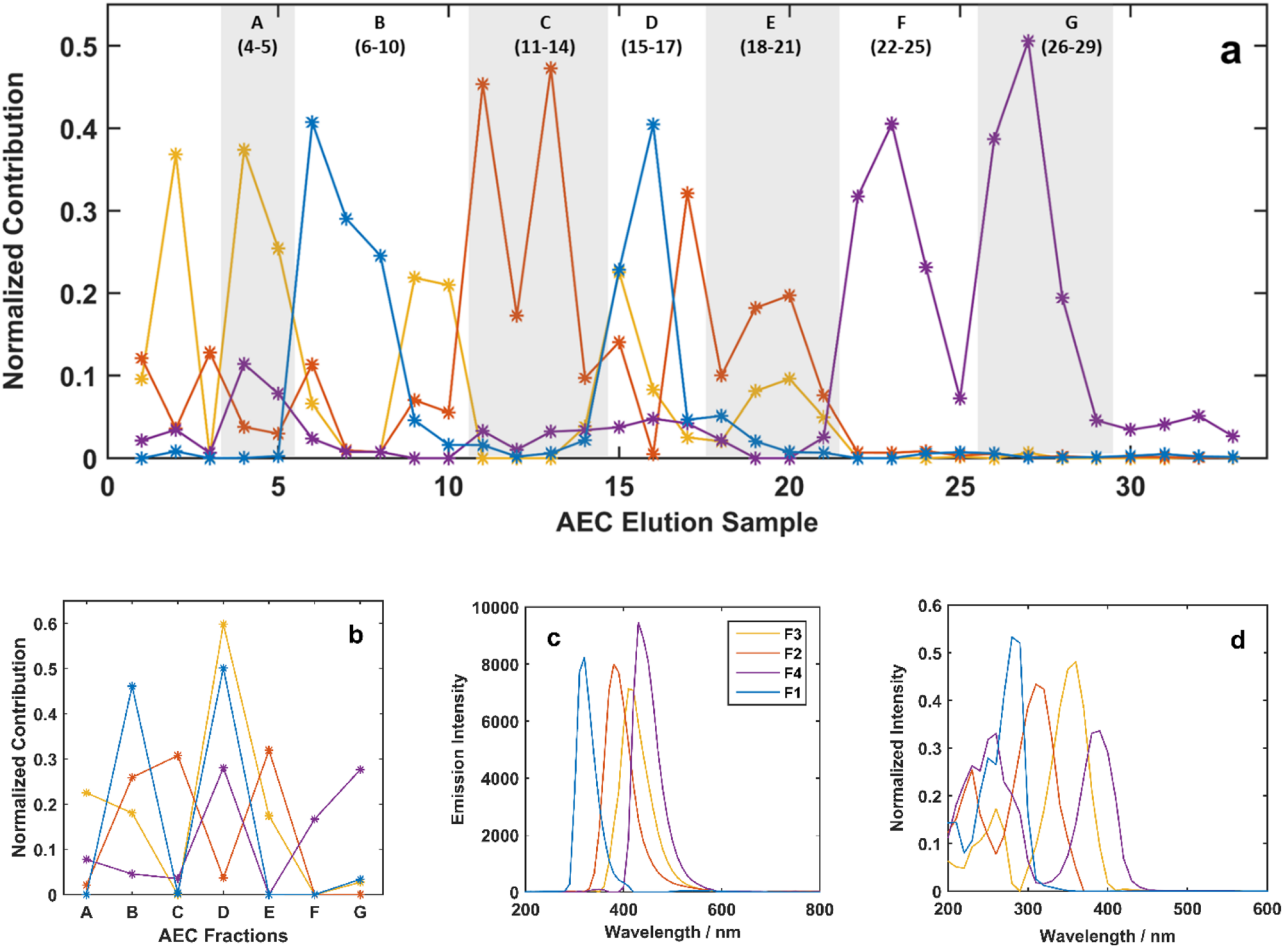
Estimated contribution (concentration) (**a**, **b**), emission (**c**), and excitation (**d**) profiles from PARAFAC analysis of EEMs of thirty-three AEC elution samples augmented with seven pooled fractions.

Seven pooled fractions were achieved through pooling the successive samples with similar EEM patterns, from the thirty-three eluted samples (Figure S3). Each pooled fraction has an almost unique EEM pattern, however, the two peaks excitation layout could be considered as a common characteristic for all seven fractions. Fraction indices of pooled elution samples are indicated in Fig. 1a. For the A and F fractions, EEM spectral pattern is independent of the excitation wavelength (Figure S3). The dependency of emission pattern to excitation wavelengths was revealed for the B and D fractions. Regarding the observed trends in the location and the pattern of EEM peaks in the pooled fractions A to G, it is hard to clearly state that there is a relation between surface charges of CNDs and their PL characteristics.

To gain quantitative information on AEC thirty-three elution samples and seven pooled fractions of the stacked three-way data were introduced to the parallel factor analysis (PARAFAC) model. A trilinear model with an explained variance of 94.6% reveals that four principal fluorophores in the CNDs mixtures are responsible for observed PL. The emission and excitation spectra of the four fluorophores are illustrated in Fig. 1b,c. Estimated concentration profiles (Fig. 1a,b) represent the relative contribution and/or evolving concentrations (chromatograms) of each fluorophore for thirty-three elution samples and seven pooled fractions in PL maps.

The chromatogram of thirty-three elution samples (Fig. 1a) reveals that CNDs fractions with different surface charges mainly contain different fluorophores. The dominating fluorophores in initial and last elution samples are the F3 and F4, which are located on the most positive and the most negative dots, respectively. CNDs containing F1 fluorophores (B and D) have more positive sites compared to dots with F2 fluorophores (C and E). The overall order of net negative charges on the surface of CNDs, regarding their fluorophore contents, could be considered as CND-F4 > CND-F2 > CND-F1 > CND-F3. Particles in A fraction with low retention time are mostly the F3 containing dots with almost the lowest negative surface charge, compared to other particles. Also, it seems that the small contribution of F4 in A fraction leads to the small number of negative charges and low retention of this group of CNDs in the column. Fluorophore labels are not assigned as F^+^, F^++^, or F^−^ because it is not certain that surface charges are mainly from fluorophores.

It is figured out that the type of fluorophore has a correspondence to the net surface charge of CNDs, which would cause the separation of CNDs mixture in AEC. F and G fractions mainly consist of F4 fluorophore, although there is a minute contribution of F3 in the G fraction. It could be concluded that some functional groups (without optical features), other than fluorophores, contribute to the surface charge on CNDs and leads to differences in the retention time of the two fractions. Reactions of precursors for the production of CNDs in elevated temperature and pressure are complex and in many different ways. So, they result in many different types of particles with many similarities and variances regarding size, fluorophores, surface functional groups, and charges. The employed ion-exchange column-based separation in this report only separated particles with similar charges in each fraction.

In general, the observed optical features (emission and absorption) of CND solution mixtures come from individual molecular by-products, supramolecular carbon clusters, and carbon core or their combinations in solution. The fractionation process by AEC along with PARAFAC helped to reduce the complexity of as-synthesized CNDs sample. This separation illustrated that fluorophores are distributed among different fractions and coexist on particles (different elution samples and fractions). As there is no common fluorophore in all eluted samples (fractions) it is a proposition that PL of CNDs rarely originated from core and core is an optically in-active structure. According to the obtained results and observed effects of the fluorophore on the surface charge of fractions, it could be concluded that CNDs are nanoparticles with an optically in-active carbon core. They carry organic functional groups and fluorophores on their surfaces that determine their optical behavior and surface charge.

### Dilution

The concentration-dependent EEM behavior of the CNDs is systematically evaluated in this section. In a process of 1:1 dilution of the as-prepared mixture of CNDs for 12 steps (with relative total concentrations of 1.000, 0.500, 0.250, 0.125, …), using deionized water, the color of CNDs solution was changed from red-brown to a transparent solution with almost no color. Figure S4 shows the excitation-emission maps of the as-prepared CNDs mixture and its stepwise dilution solutions.

**Figure 2:**
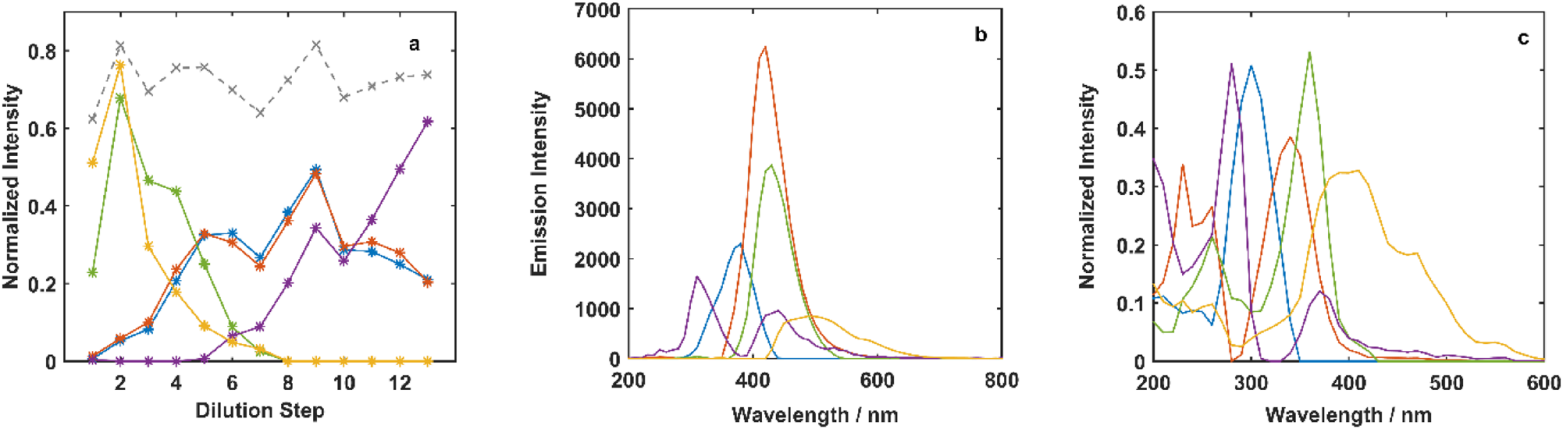
Calculated relative intensities during dilution (**a**), in addition to emission (**b**) and excitation (**c**) profiles obtained from the application of PARAFAC (98.4% explained variance) with five factors on the data from the dilution process. The gray dashed line in part (**a**) shows the normalized total variation of EEM in dilution steps.

The AEC section revealed the multicolor nature of CNDs by illustrating them as particles with multiple luminescence centers. The existence of multicolor emission in higher concentrations is mainly due to the energy transfer and quantum confinement effect that came from interactions and aggregation between CNDs. The CNDs at the high concentration tend to interact and aggregate, which increases the rate of energy exchange between particles. The increasing of total emission intensity by the first step of dilution confirms the high rate of energy transfer in form of self-absorption in the initial concentrate mixture. In the fourth step of dilution, some peaks emerge in the far blue (< 400 nm) region, which was quenched in the initial mixture. On the other hand, the peaks at green and red (500–650 nm) regions for the initial mixture decrease during dilution. The grey dash line depicted in Fig. 2a shows the total PL signal variation through the dilution that was expected to be decreasing through the dilution, although not considerably changed.

To follow the EEM changes during dilution steps in a quantitative way and to track the appearing and quenching fluorescence peaks, stacked EEMs were subjected to the PARAFAC. The resolved profiles by PARAFAC are illustrated in Fig. 2.

Figure 2a elucidates the trends for the appearance and disappearance of fluorescent components during the dilution process. Corresponding emission and excitation spectra are shown in Fig. 2b,c, respectively. Concentration profiles in Fig. 1 show the fluorophores contribution in the eluted samples and the pooled fractions, while Fig. 2a illustrates their portion in EEM spectral evolution during the dilution process. The number and shape of the resolved excitation and emission profiles for fluorophores in the dilution experiment are different from those for fluorophores in the AEC separation experiment. It means although the existing overall fluorescent groups on all CNDs in the system are the same for both experiments, the linear combinations of the signals (fluorophores) from fluorescent groups observed in the two experiments with distinct conditions are different. The initial rise in the total emission intensity of the solution is resolved into green and yellow profiles (Fig. 2a). Through the first step of dilution (mixture 1 to mixture 2), the fluorescence intensity of the solution enhances, that is due to the reduction of self-absorption and inner filter effect. Decrease and diminishment of the two profiles through the dilution steps confirm that they are made up of aggregation and are from energy acceptor CNDs. The trigger energy (excitation profile) for the yellow component (Fig. 2c) consists of a wide range of wavelengths from 200 to 600 nm, which is proper for receiving energy from many higher energy states.

Increasing for the red and blue profiles is observed as well as green and yellow in the first 2 dilution steps (Fig. 2a). For the next three dilution steps (3, 4, and 5) the red and the blue profiles, that are related to energy donor particles, grow sharply. This is due to the dissociation of aggregated particles and reduction of energy transfer during dilution. This rising is along with the decreasing of yellow and green profiles (that correspond to energy acceptor particles) and demonstrates the getting apart of CNDs. By progressing the dilution to steps 6 and 7, the rising violet profile (that is from energy donor CNDs) with higher emission energy (310 nm), leads to a decrease in the intensity of other profiles. The next level for dissociation of aggregated CNDs happens in steps 8 and 9 in which, the green and yellow profiles tend to zero and the others ascend sharply. In steps 10 to 13 of dilution, the dilution results in a decreasing trend for the red and blue profiles but an obvious uprise in the violet profile. The violet profile consists of some luminescence states including 310 nm, 440 nm, and the broadband at 500–600 nm, which mainly forms the EEM pattern in the last diluted solution (Figure S4). The CNDs that are related to the red and the blue profiles are energy donors to the particles that correspond to yellow and green profiles. Additionally, they are energy acceptors from particles that are related to the violet profile. It was concluded from the observed rise and fall off of the red and the blue profiles along with the experiment steps. During the dilution experiment, the overall emission intensities of CND mixtures (grey dashed line) remained almost steady although the emission wavelengths are changed. This is another piece of evidence that confirms the presence of energy transfers between particles.

### Trajectory pattern

For juxtaposition and classification of the fractions separated from the AEC column and the mixtures obtained from dilution steps, patterns and shapes of their informative second-order EEM fluorescence images were considered. To make the comparison of large complicated image matrices possible, emission trajectory patterns were considered. The three-way data of stacked EEM fluorescence from the seven fractions and the thirteen mixtures of dilutions were unfolded to a large matrix. Scores for each set of data obtained by applying principal components analysis (PCA) on the large matrix. Scores scatter plot of the two principal components for each fraction or dilution step forms a trajectory line. Points in a trajectory line represent columns in an EEM matrix. Utilizing trajectory lines makes it possible to simply illustrate several matrices in a two-dimensional plot.

**Figure 3:**
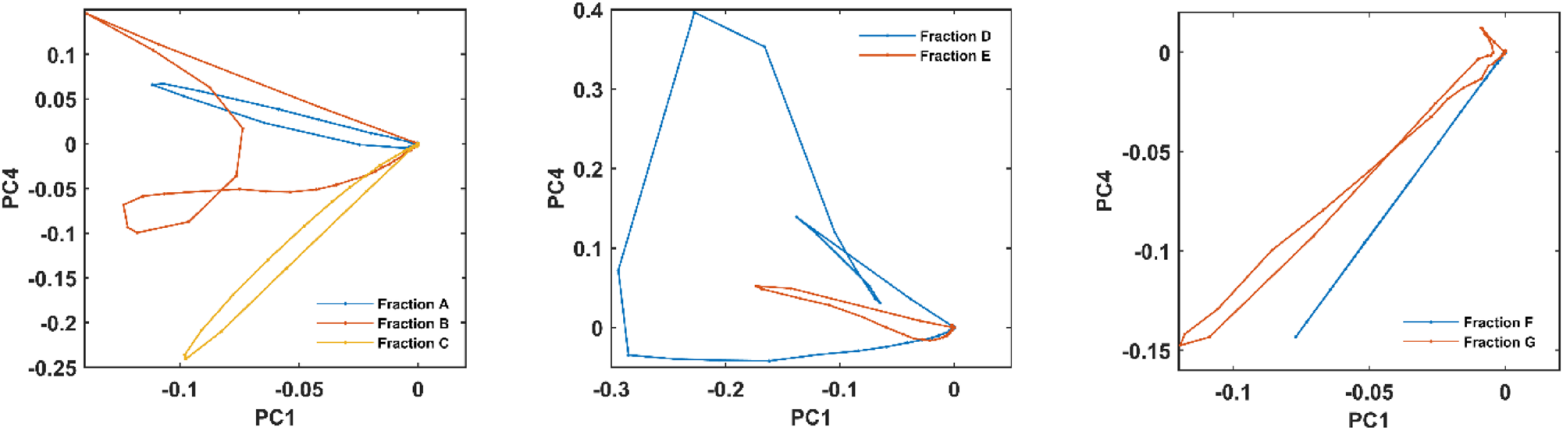
Trajectories for the seven EEM fluorescence images from pooled AEC fraction of CNDs mixture.

Emission trajectories for seven fractions of AEC are illustrated in Fig. 3. The patterns for all seven fractions are unique and each one is in a specific form and direction. The trajectories for B and D fractions show broader patterns, comparing to the others. Since PCA seeks the main variation patterns in the data, these trajectories with distinctive patterns reveal that each fraction is principally composed of various types and amounts of emitting groups. The contribution of all four fluorophores in B and D fractions (Fig. 1b) confirms their broad trajectory patterns (Fig. 3). The A, C, E, and G fractions with simple and narrow trajectory patterns consist of one or two dominating fluorophore(s). The simplest pattern among seven fractions belongs to the F (a line passing through the origins), the almost pure fraction containing one dominating fluorophore.

**Figure 4:**
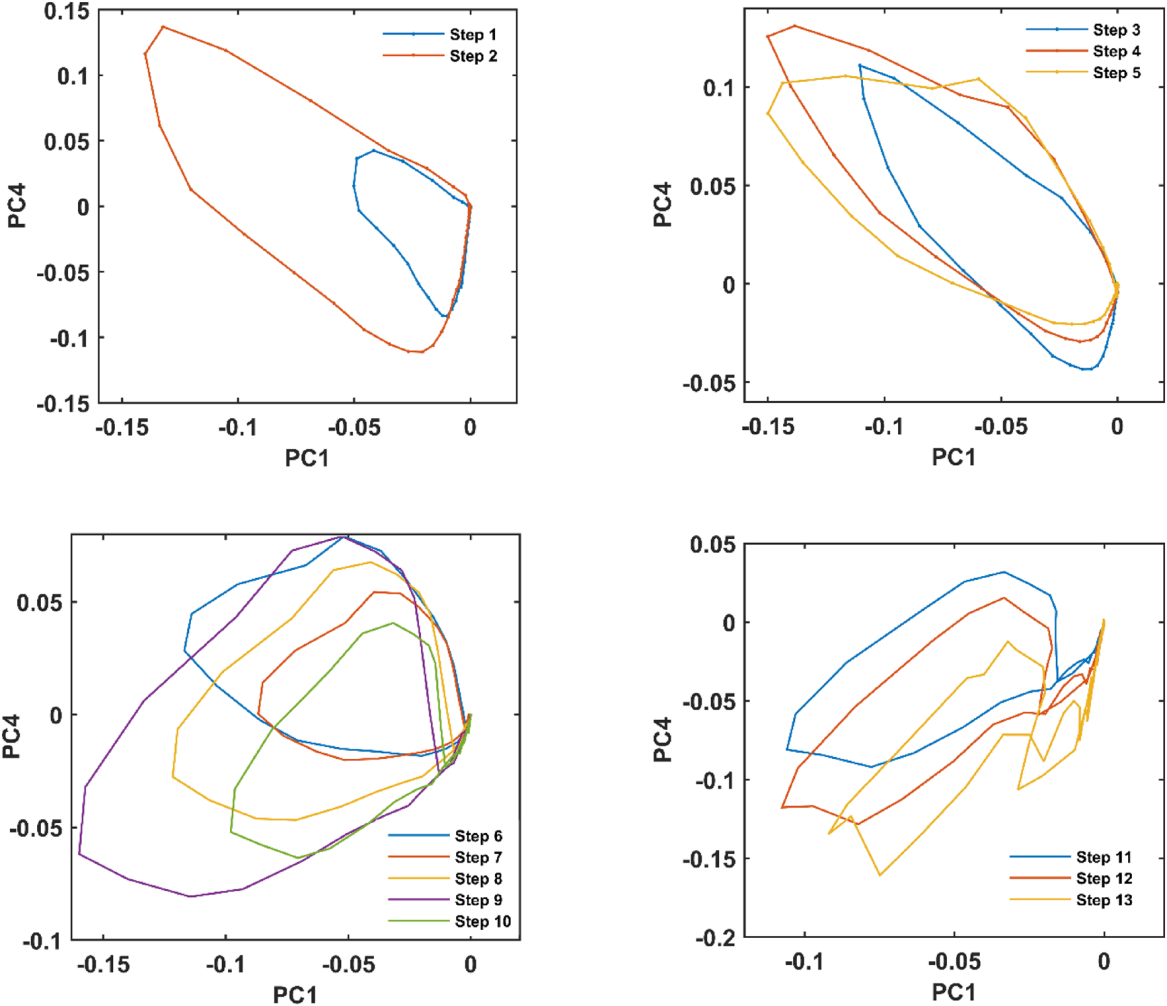
Trajectories for the thirteen EEM fluorescence images from different steps of dilution of CNDs mixture.

According to Fig. 4, there are more similarities in the emission trajectories of the dilution data, compared to that from AEC. The unique trajectory pattern of the first step is followed by a wide pattern for the second step which shows the increase in EEM signal variation (Figure S4). For the third to the fifth step (Fig. 2a), due to the appearance of new fluorescent components, there are similar trajectory patterns that reveal the small changes in the EEM characteristics of the solutions. The violet profile (Fig. 2a) rising at the sixth step, leading the shape of emission trajectories for the following steps. The total decrease of EEM fluorescence intensity in the seventh step resulted in its smaller pattern, compared to that in the sixth step. The evolution of patterns was almost finished at the eighth step, and after that, there are similar emission trajectories for the dilution process. These similar patterns reveal that the EEM fluorescence structure of diluted CND mixtures remained almost identical for the last steps of dilution.

**Figure 5:**
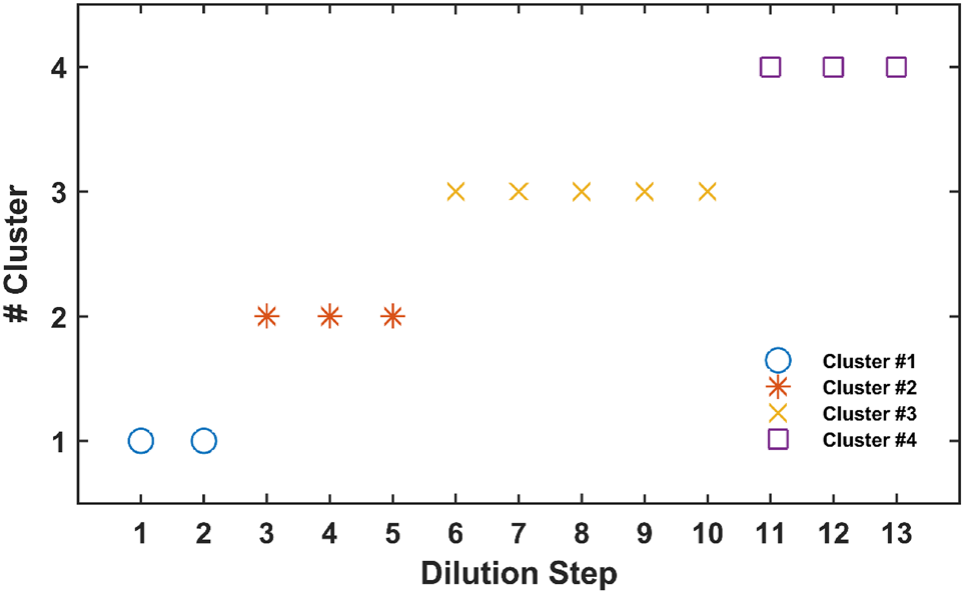
Resulting classes from the application of *k*-means clustering for dilution series data.

In addition to the visual comparison, the similarities and differences in trajectory patterns were quantified through the *k*-means clustering algorithm. The point is that *k*-means is useful for clustering samples that are vectors (first-order samples), and points in the data space. Now the considered samples are trajectories (second-order samples), each of which is a matrix and some points in data space. To make proper input for *k*-means, a matrix of distances between trajectories was formed and resulted in a matrix including a vector for each trajectory. The rows of the 1^st^ order matrix of trajectories related to mixtures from all the thirteen steps of dilution were separated into four clusters (Fig. 5). Mixtures from the two first steps formed a cluster as it is was expected from their profiles in Fig. 2a, obtained by PARAFAC. The second cluster involved the next three steps (3, 4, and 5) that are along with the rising of blue and red profiles. The rising of violet profile in the sixth step took the lead in trajectory patterns for the next five steps being grouped in the third cluster. At last, the decrease in the blue and red profiles after the tenth step made the fourth cluster with three members.

### Infrared spectra

As it is stated and confirmed in the AEC section, the CNDs mixture involved different organic molecules as well as fluorophores. The relative contribution of four fluorophores in the CNDs mixture was resolved by PARAFAC from unpooled and pooled AEC fractions. The fluorescence data only consist of information about molecules with emission properties. Whereas, measured Fourier transform infrared (FT-IR) spectra contain the information about all molecules in CNDs, either emitting or non-emitting.

The FT-IR spectra of seven pooled fractions (A, B, C, D, E, and F and G) of CNDs are illustrated in Figure S5. Some bands are almost common for all fractions, such as broad bands at 3050–3650 cm^−1^ from v(O–H) and v(N–H), s(N–H) at 1570 cm^−1^, carboxylic acid s(O–H) at 2400 cm^−1^, and bands at 1650–1780 cm^−1^ due to the s(C=O).

The fingerprint region (from about 1500 to 500 cm^−1^) of the FT-IR spectra shows that CNDs in the A, B, and C regions contain similar combinations of functional groups, although the dominant fluorophores in the regions are different. Similar IR spectra of A, B, and C shows the similarity of the organic structure of CNDs that contain F1, F2, and F3 fluorophores, although their EEM fluorescence spectra are different. The consecutive retention of A, B, and C fractions to the cationic column is confirmed by the increase of broadband at 2400 cm^−1^ attributed to OH groups of carboxylic acids and decreasing the intensity of the N–H peak by about 3450 cm^−1^. The D fraction has a quite different pattern from the three previous fractions. The addition of NaCl to the mobile phase leads to reducing the band ascribed to the carboxylic acids in the D fraction. The carboxylic acid peak for the E fraction has raised along with decreasing in nitrogen groups and specific fingerprint region, compared to the other fractions. For the F and G fractions, similar emission properties (Fig. 1b) resulted in a similar pattern in the FT-IR spectra. While, the broadband from O–H in carboxylic acids is suppressed in F portions due to the presence of a higher concentration of Na^+^ in the mobile phase and substitution with H^+^ in carboxylic acids, as COO–Na^+^.

To obtain a proper estimation of the pure FT-IR spectra of the four fluorophores, the relation between the measured FT-IR spectra in seven different fractions and the corresponding calculated relative concentration of each fluorophore by PARAFAC was considered. In the classical approach of linear regression, the FT-IR intensities were treated as the dependent variables and the calculated relative concentration profiles as the independent variables, which resulted in estimating the pure FT-IR spectra of the fluorophores. It is worth noting that the four fluorophores would have many spectral similarities regarding IR active functional groups, and as a result, estimated pure IR spectra from a four components regression model were not chemically acceptable.

**Figure 6:**
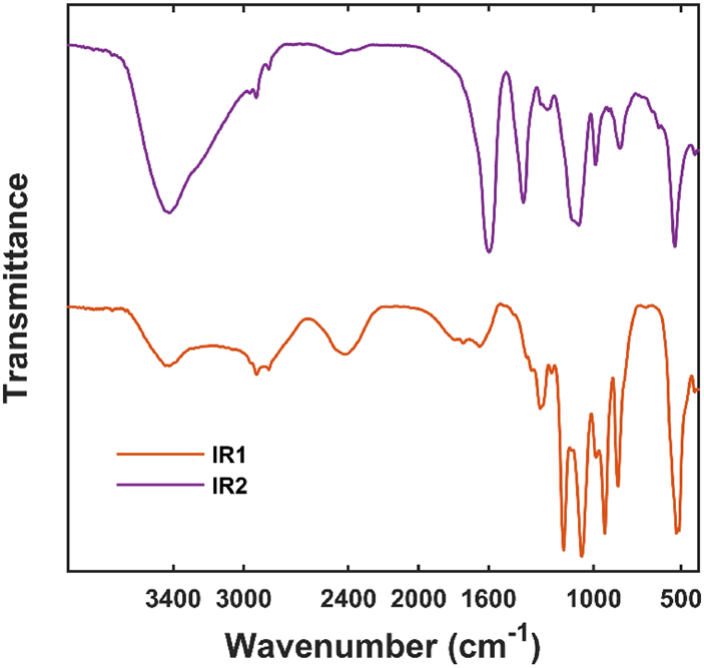
Estimated pure FT-IR spectra for CNDs containing IR1 and IR2 fluorophores.

By examining different sets of the concentration profiles in regression analysis, only two pure FT-IR spectral profiles (IR1 and IR2) were properly estimated from the model. The two chemically acceptable pure FT-IR spectra from the regression model are illustrated in Fig. 6. This means that pure FT-IR spectra of CNDs are linear combinations of these two IR spectra. Regression analysis reveals that although their PL spectra are completely different, the pure IR spectra and functional groups' content of CNDs that contain different fluorophores are highly similar and are in two categories. Although the IR spectra of each fraction are made by a linear combination of these two spectra, the IR2 spectrum is more similar to the IR spectra of F and G fractions (Figure S5) and IR1 could be considered as representative of the first five fractions. Comparability of the estimated pure IR spectra is due to the presence of amine, alcohol, alkane, carboxylic acid, and carbonyl groups in both profiles (Fig. 6). On the other hand, the signals for carbonyl groups (1600–1800 cm^−1^ and about 1400 cm^−1^) in IR2 are an obvious difference between these two spectra. The two estimated pure FT-IR spectra could explain about 90 percent of the information in FT-IR data, which reveals the presence of similar functional groups and distinct photo-illuminating molecules (fluorophores) in carbon nano-dot mixtures.

## Conclusion

The hydrothermally synthesized CNDs mixture resulted in a luminescence solution, with relatively excitation-dependent emission wavelengths. The existence of different aggregation stages and energy transfer between and within CNDs, as the function of concentration, was revealed through a simple dilution experiment along with detailed data analysis. PARAFAC illustrated the individual progress and diminish of different luminescence centers during dilution and the trajectory analysis laid out the similarities among the spectral behavior of diluted samples. The fractionation of CNDs by their surface charges showed that luminescence characteristics were changing through the elution samples. CNDs consist of different luminescence centers contains different surface charges, although the relation of the surface charges in CNDs to functional groups is not clear. The FT-IR analysis also reveals the presence of carboxyl and nitrogen-containing functional groups in CNDs and their structural similarities.

## Methods

Synthesis of CNDs mixture was performed hydrothermally at 180 °C for 4 h by using citric acid and 2,3-diaminopyridine as sources of carbon and nitrogen, respectively. The high-resolution transmission electron microscopy (HRTEM) was used to investigate the structural characterization of prepared CNDs. The majority of particles show less than 10 nm size (Figure S1). The series dilution of CNDs was done by deionized water by a ratio of 1:1. At every step of dilution, a specific volume of carbon dots solution was completely blended with deionized water and then the matrix of excitation-emission fluorescence was measured to follow the PL changes during dilution. A glass column packed with the diethylaminoethyl (DEAE) Sepharose anion exchanger was used for fragmentation of CNDs mixture to several parts with alike surface charges. The packed column was washed and conditioned by the phosphate buffer solution (pH = 7.0) before introducing of carbon dots mixture. The elution of the column was done at the rate of approximately 1 mL/min through the mobile phase (phosphate buffer solution, pH = 7.0). The sodium chloride was increased gradually (0.1 M to 1.0 M) in the buffer solution for increasing the ionic strength of the mobile phase. In-detail analysis of fluorescence data for dilution series and chromatographic fractionation were performed using PARAFAC and *k*-means clustering of trajectory patterns. PARAFAC is a powerful chemometrics method to deconvolution of data with a dimension higher than two. PARAFAC resolves the patterns in each dimension under a predefined constraint named trilinearity. This constraint assumes that a set of specific factors define the variation of data through some weights. Another constraint like nonnegativity could be added based on the nature of data. Explanation about PARAFAC is in the supplementary information part. A simple and popular method for partitioning data with *n* observations into a *k* fixed beforehand clusters is *k*-means clustering, in which each observation belongs to the cluster with the nearest *mean*. A cluster refers to a collection of data points aggregated together because of certain similarities, without supervision.

## Supplementary Information

Supplementary Information.
