# Supplementary material for: Perception on aggregation induced multicolor emission and emission centers in carbon nanodots using successive dilution, anion exchange chromatography, and multi-way statistics
Source: Sci Rep. 2021 Jul 7;11:13996. doi: 10.1038/s41598-021-93212-w (PMC8263574; doi:10.1038/s41598-021-93212-w)

Perception on Aggregation Induced Multicolor Emission and Emission Centers in Carbon Nanodots Using Successive Dilution, Anion Exchange Chromatography, and Multi-Way Statistics

Mohsen Kompany-Zareh[a,b]*, *Saeed Bagheri*[a]

[a] Department of Chemistry, Institute of Advanced Studies in Basic Sciences, Zanjan, 45137-66731, Iran.

[b] Trace Analysis Research Centre, Department of Chemistry, Dalhousie University, P.O. Box 15000, Halifax, NS B3H 4R2, Canada.

*kompanym@iasbs.ac.ir

Parallel Factor Analysis (PARAFAC)

The fluorescence data arranged in $I \times J \times K$ three-way array. The first index (*I*) refers to the samples, the second (*J*) to the emission wavelengths, and the third (*K*) to the excitation wavelengths (Scheme S1).

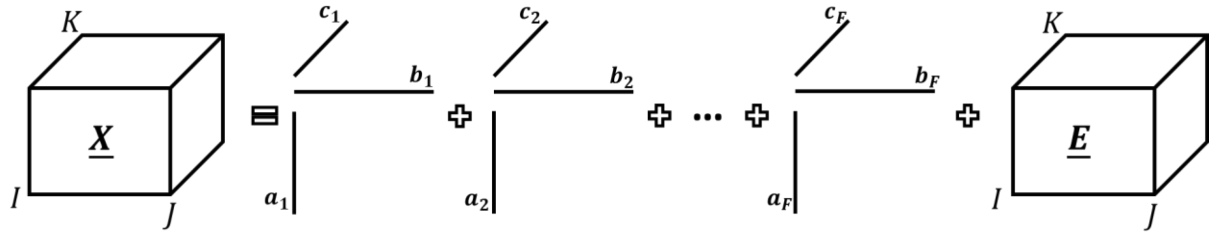

Scheme S1. Graphical representation of F-factor PARAFAC model of the data array $\underline{\boldsymbol{X}}$.

PARAFAC model the data according to the below equation:

$$x_{ijk} = \sum_{f}^{F} a_{if} b_{jf} c_{kf} + e_{ijk}$$

$$i = 1.\dots.I\ ;\ j = 1.\dots.J\ ;\ k = 1.\dots.K$$

where x_{ijk} is the intensity of fluorescence in an *i*th sample at *j*th emission and *k*th excitation wavelengths. The *F* value shows the number of factors or in this case number of fluorophore analytes. The elements in a_{if} could be interpreted as the relative concentration of analyte *f* for the *i*th sample. The $\boldsymbol{b}_f$ with elements b_{if} $(i = 1.\dots.I)$ is the estimated emission spectrum of this analyte and likewise $\boldsymbol{c}_f$ is the estimated excitation spectrum. The optimum number of analytes (*F*) determines through the meaningfulness of the estimated profiles (**a**, **b,** and **c**) according to the prior Physico-chemical knowledge and structure of the noise array ($\underline{\boldsymbol{E}}$). The elements in $\underline{\boldsymbol{E}}$ should be as minimum as possible while **a**, **b** and **c** are logical.

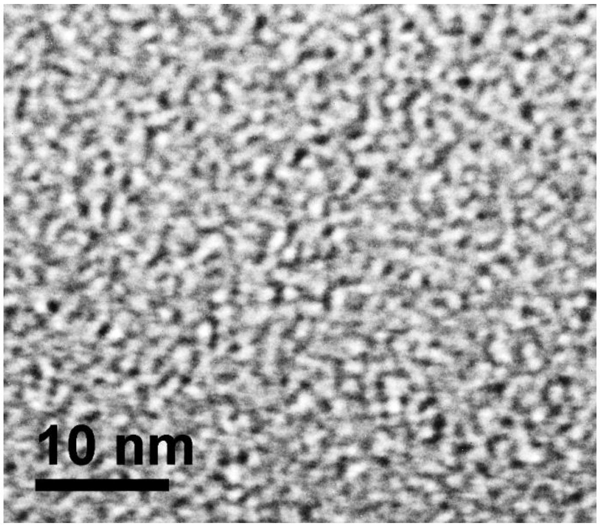

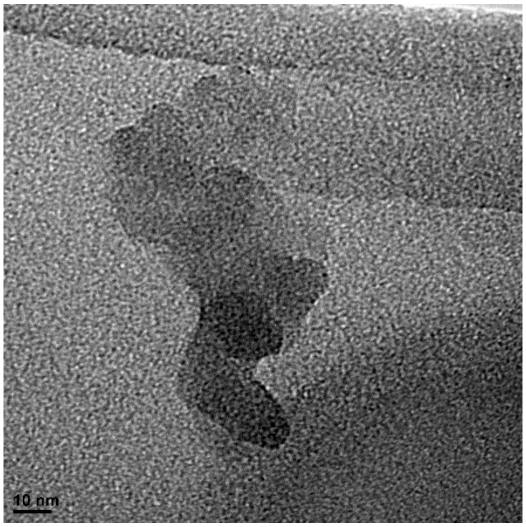

Figure S1. HRTEM of as-prepared carbon nanodots

Figure S2. Emission-Excitation map of some elution samples through AEC.

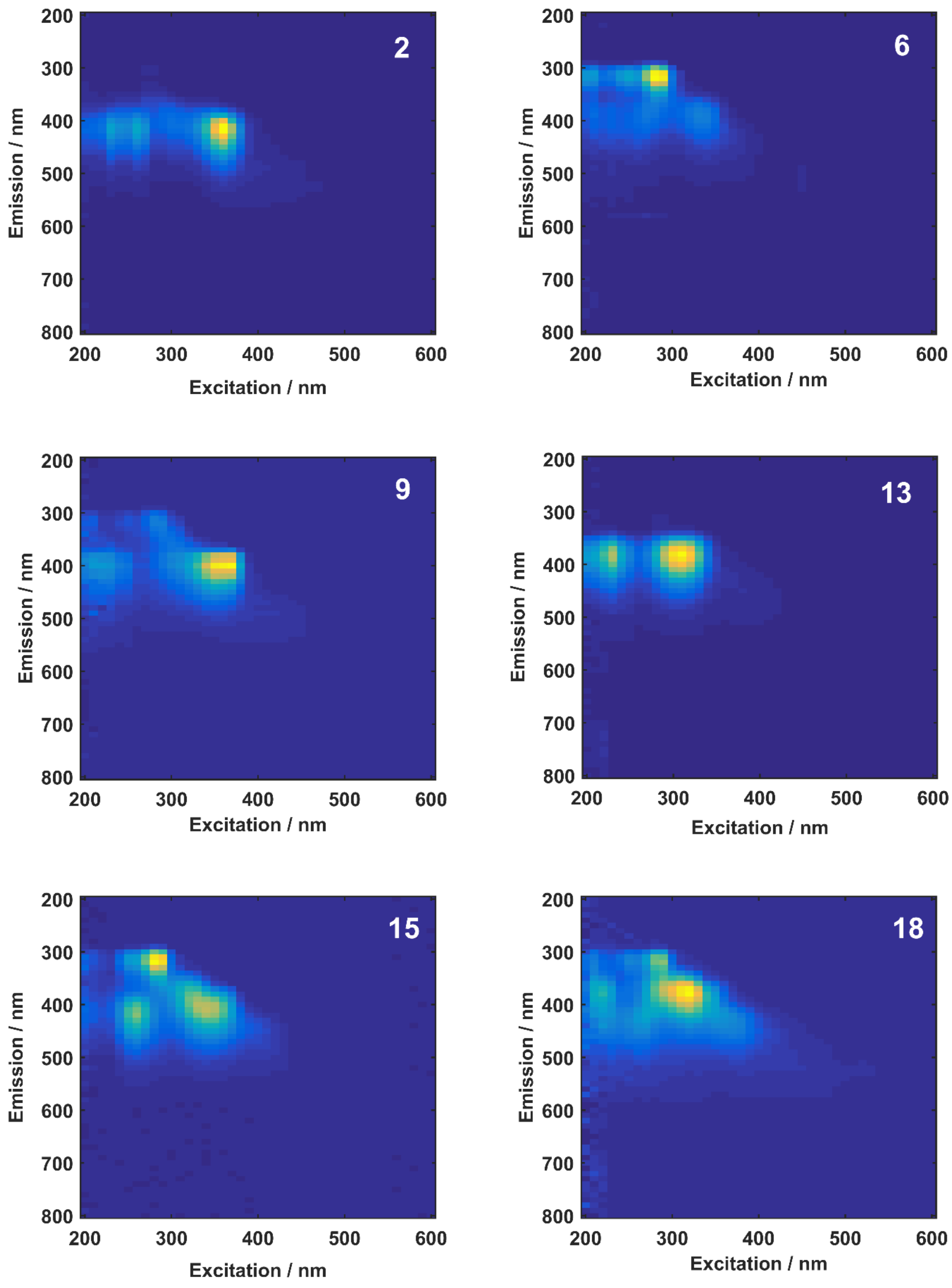

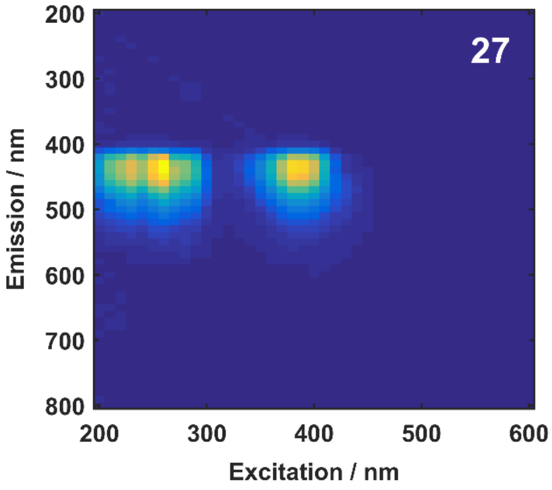
27
Emission / nm
200
300
400
500
600
700
800
Excitation / nm
200
300
400
500
600

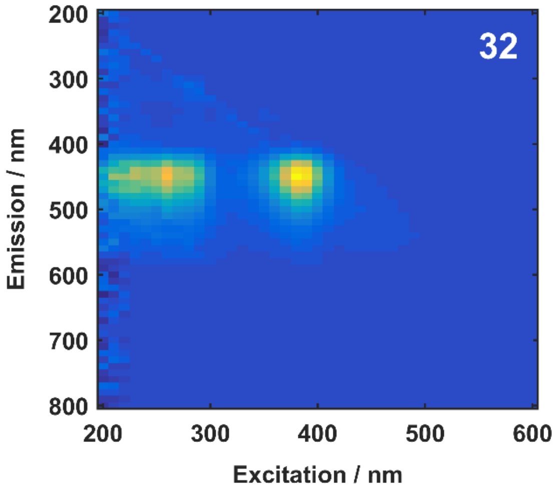
32
Emission / nm
200
300
400
500
600
700
800
Excitation / nm
200
300
400
500
600

Figure S3. Emission-Excitation map of 7 pooled fractions through AEC.

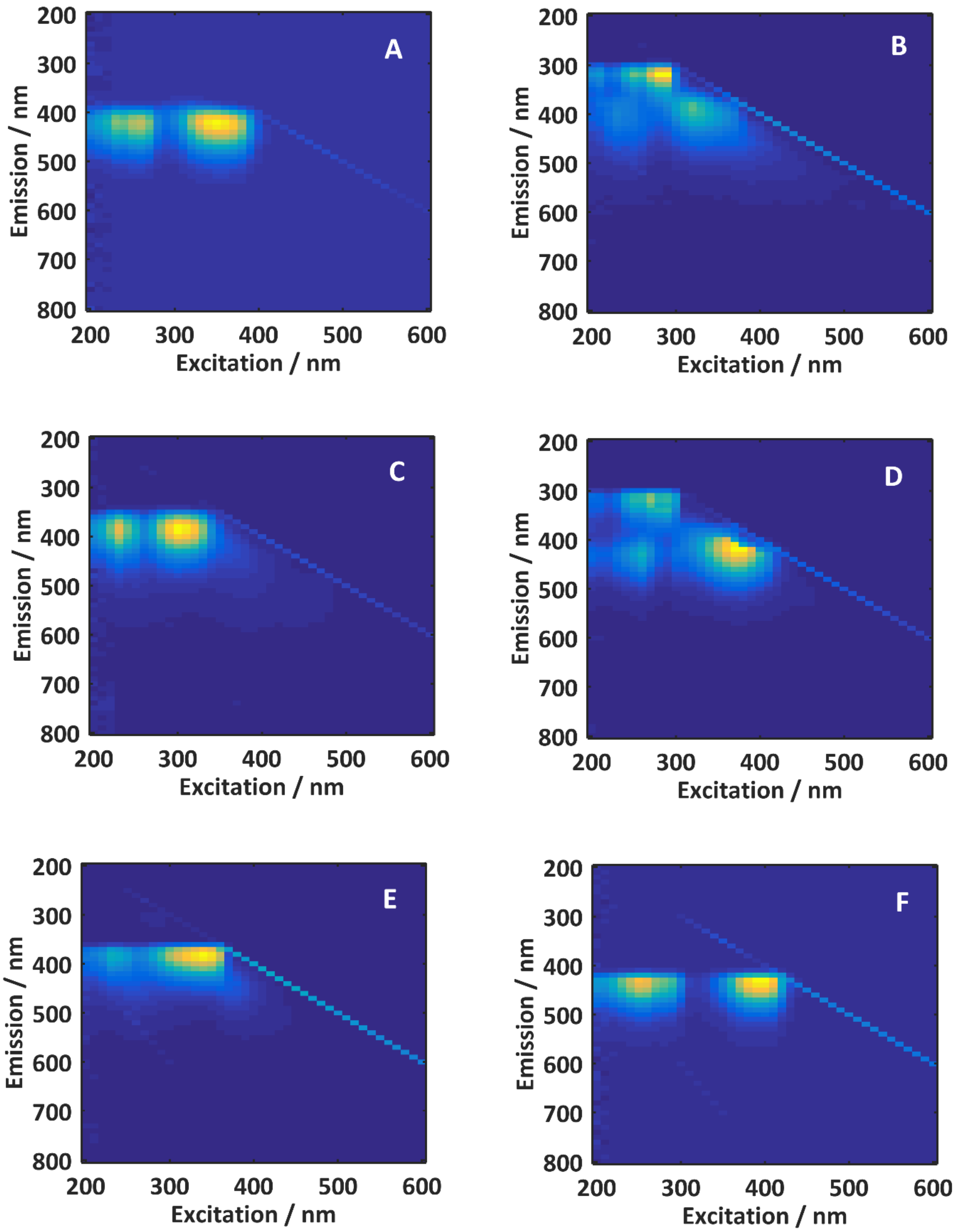

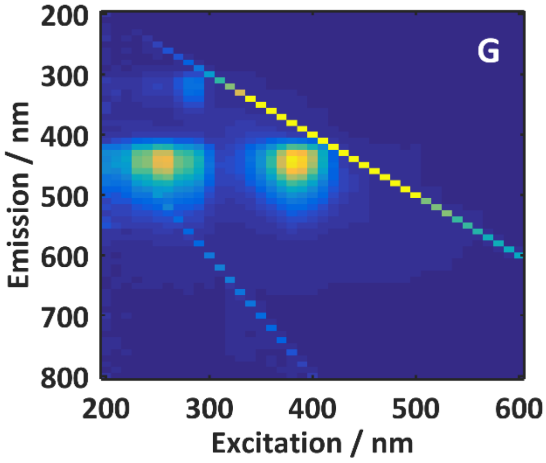
G
Emission / nm
Excitation / nm
200
300
400
500
600
700
800
200
300
400
500
600

Figure S4. Emission-Excitation map of stepwise dilution of as-prepared CNDs

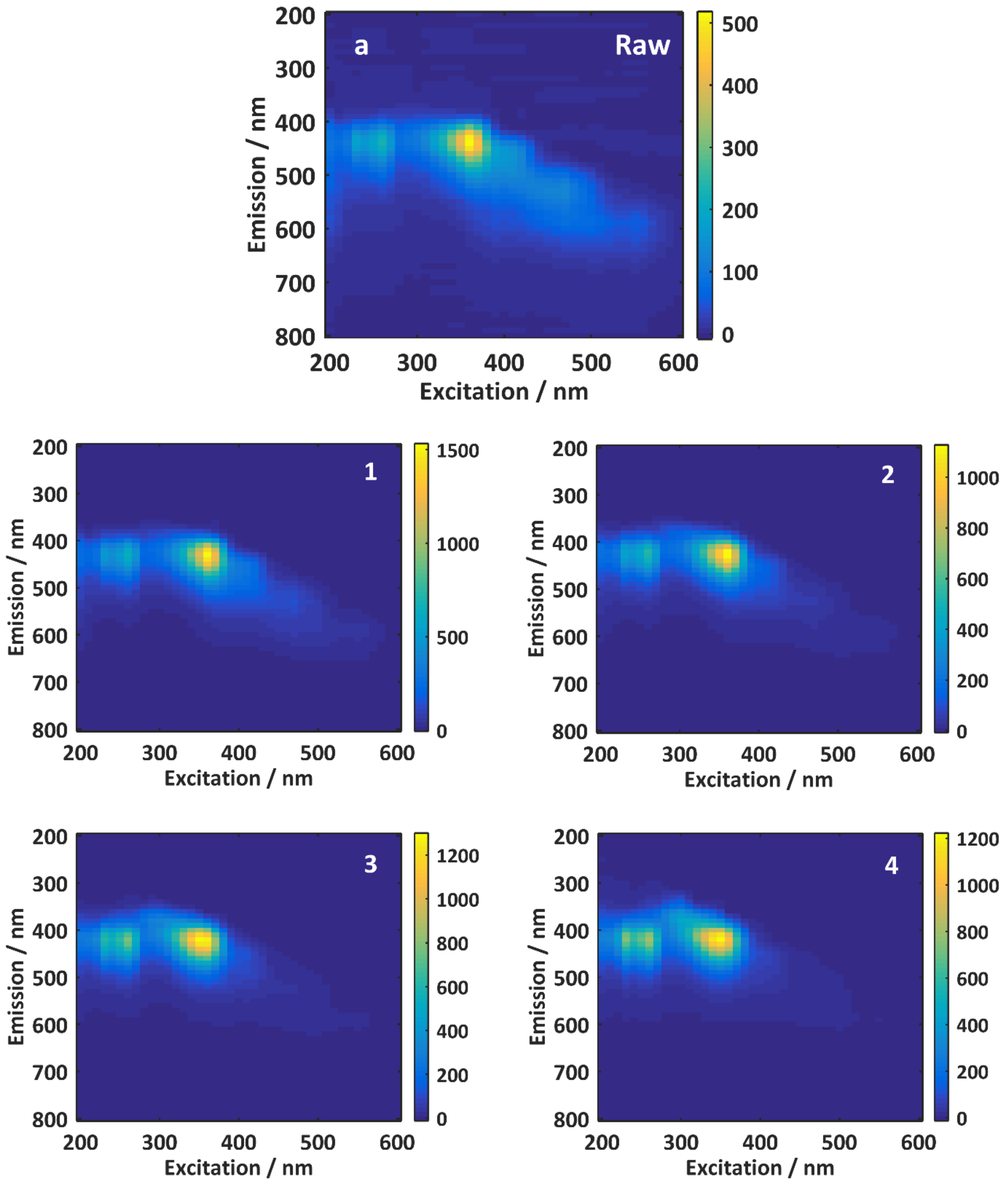

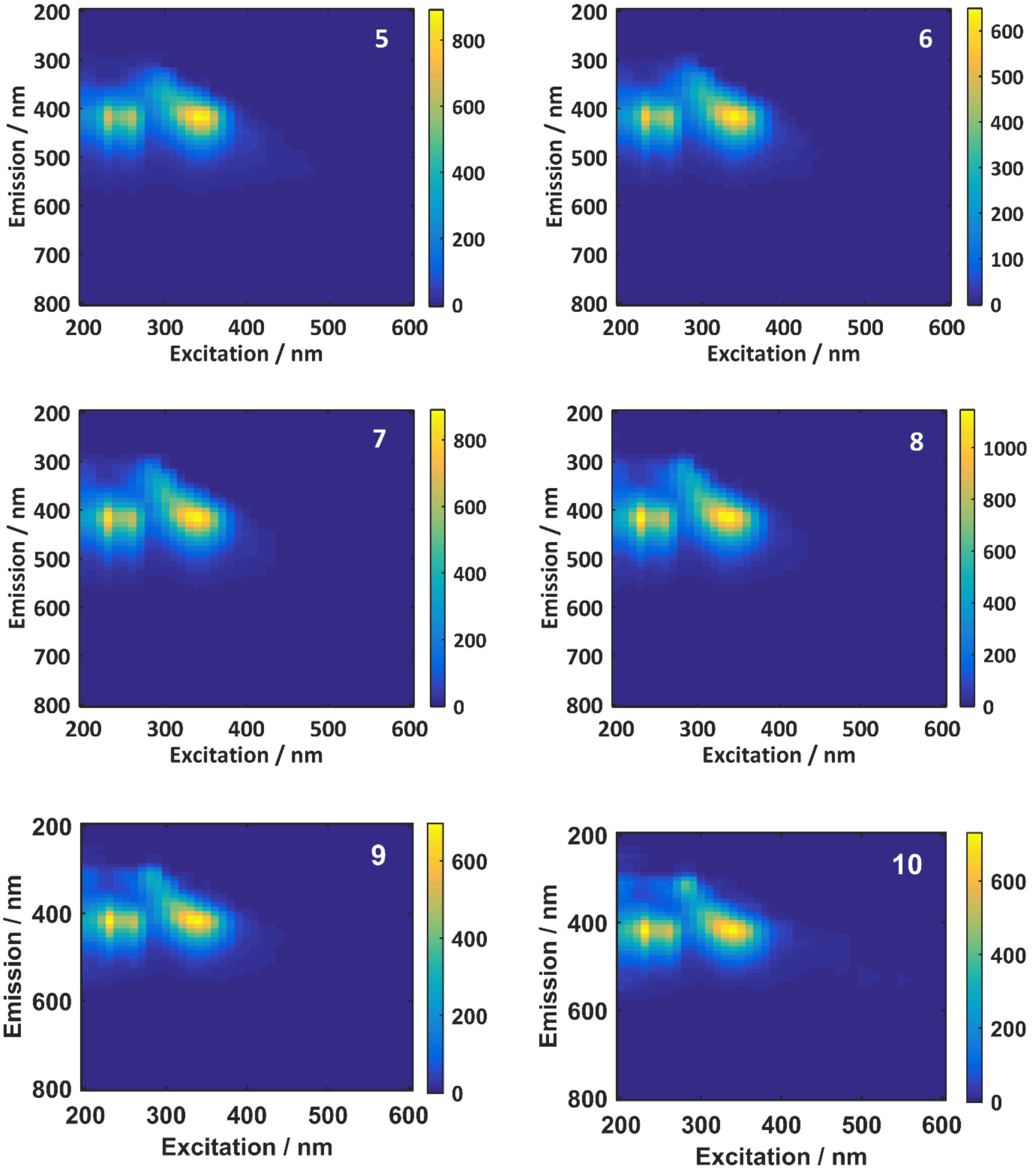
5
6
7
8
9
10
Emission / nm
Excitation / nm
200
300
400
500
600
700
800
0
100
200
400
600
800
1000

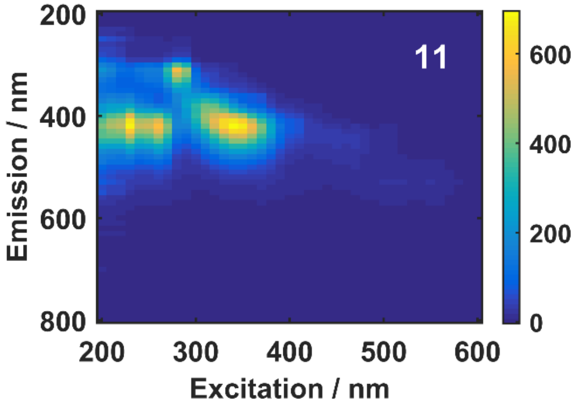
11
Emission / nm
200
400
600
800
Excitation / nm
200
300
400
500
600
0
200
400
600

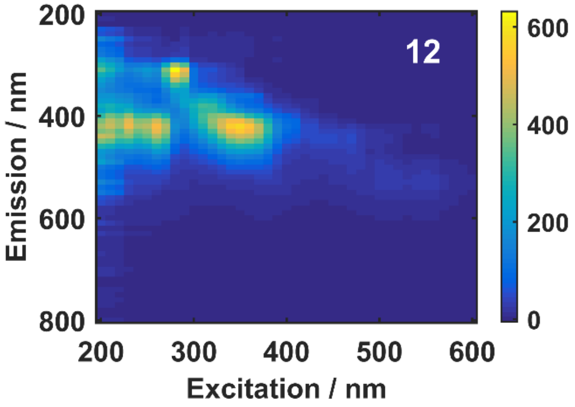
12
Emission / nm
200
400
600
800
Excitation / nm
200
300
400
500
600
0
200
400
600

Figure S5. FTIR spectra of the seven selected CND samples

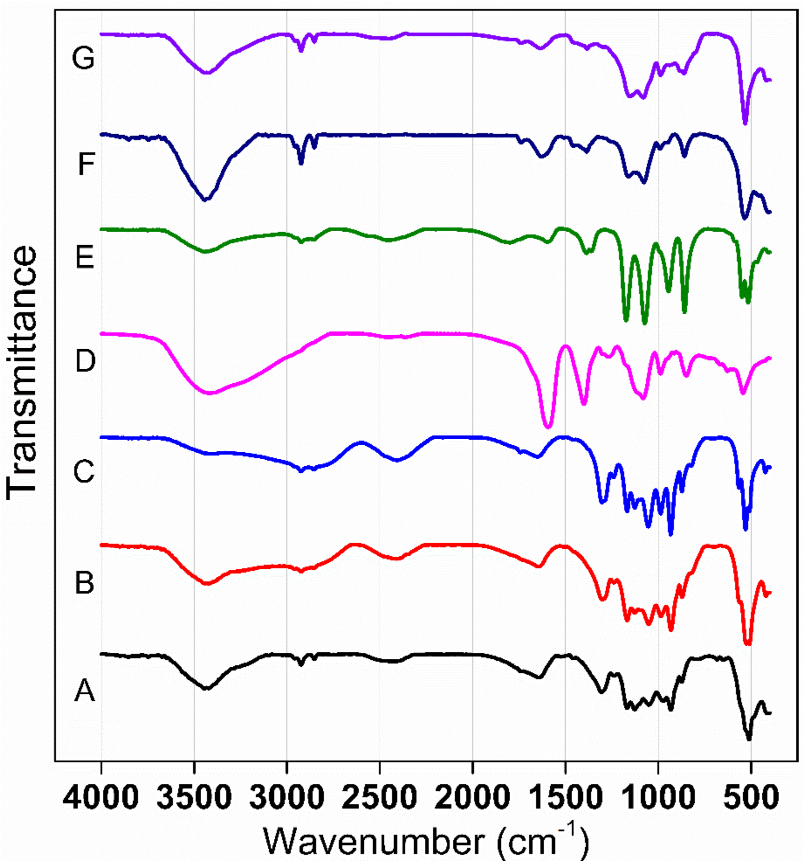

Supplement: Supplementary file 1 — Supplementary Information. [file 41598_2021_93212_MOESM1_ESM.pdf]
